# Prevalence of symptoms and their association to health-related quality of life among older men in Sweden – a cross-sectional study

**DOI:** 10.1186/s12877-026-07152-z

**Published:** 2026-02-10

**Authors:** Emma Sjöberg, Slavica Kochovska, David C. Currow, Magnus Ekström, Max Olsson

**Affiliations:** 1 Sydnärke Public Health Team, Lekeberg Municipality, Lekeberg, Sweden; 2 Flinders University, Adelaide, Australia; 3 Faculty of Medicine, Department of Clinical Sciences Lund, Respiratory Medicine, Allergology and Palliative Medicine, Lund University, Wigerthuset, Remissgatan 4, Lund, SE-221 84 Sweden; 4 Faculty of Health Science, Department of Public Health, Kristianstad University, Kristianstad, Sweden

**Keywords:** Symptoms, Older population, Health related quality of life

## Abstract

**Background:**

Symptoms can negatively impact health-related quality of life (HrQoL) in older adults, yet data on their prevalence and impact in older men are limited. This study aimed to examine the prevalence of nine core symptoms and their associations with HrQoL in older men.

**Methods:**

A cross-sectional, population-based study was conducted among 907 men aged 73 years in Sweden. Participants rated nine symptoms on a 0–10 scale: pain, tiredness, sleepiness, nausea, loss of appetite, depression, anxiety, shortness of breath, and overall well-being. Symptoms were considered present if scored ≥ 1. HrQoL was measured using the physical and mental component scores of the Short-Form 12 Health Survey (SF-12v2). Associations were analyzed using linear regression adjusted for confounders.

**Results:**

Among participants, 16% reported respiratory diseases and 35% cardiovascular diseases. The most common symptoms were tiredness (78%), poor well-being (77%), and pain (73%). Depression and anxiety were reported by 46% and 32%, respectively. The strongest associations between symptoms and physical HrQoL were (beta coefficient; 95% confidence interval): pain (-2.05; -2.25 to -2.24), tiredness (-1.98; -2.22 to -1.74), and shortness of breath (-1.73; -1.97 to -1.49) and for mental HrQOL: depression (-3.50; -3.74 to -3.27), anxiety (-3.40; -3.70 to -3.11), drowsiness (-2.35; -2,64 to -2.07), and tiredness (-2.30; -2.56 to -2.03).

**Conclusion:**

Symptoms are highly prevalent among older men and significantly associated with lower HrQoL and pain, tiredness, depression, and anxiety were the most impactful. These findings underscore the importance of targeted symptom screening and management to promote healthy aging and well-being.

## Introduction

With demographic shifts leading to a substantial increase in the older population globally [1], understanding factors that contribute to health-related quality of life (HRQoL) among older individuals becomes of vital importance [2]. HRQoL bridges the gap between health and quality of life, measuring how health influences overall well-being and vice versa [2]. HRQoL is a key aspect of life and plays a crucial role in understanding the dynamics of aging and its impact on individuals and societies (WHO, 2022).

Chronic health conditions, multi-morbidity and a high burden of symptoms are prevalent in the older population and are strongly associated with decreased HRQoL [3]. Common symptoms such as pain, tiredness and breathlessness can significantly limit daily functioning and are associated with lower HRQoL [4, 5]. Depression and anxiety are also more prevalent in older people and among those with chronic health conditions; both are known to be associated with significantly reduced HRQoL [6]. In men, depression and anxiety may be under-reported or interpreted as a natural part of aging, contributing to under-diagnosis and lack of support [7]. Yet, impaired mental health can lead to reduced participation in social life and increase the risk of isolation and inactivity [8].

There is a lack of studies examining the prevalence of multiple symptoms and compared their association to HRQoL in the older population [9]. Pain and fatigue are especially related to worse HrQoL among cancer patients [2], and breathlessness among people with pulmonary diseases [3]. Among patients with diabetes and heart disease, especially depressive symptoms were related to worse HrQoL [4]. However, the studies we found have either focused on specific health conditions or examined single symptoms in isolation, rather than exploring the overall symptom burden and its relationship to HRQoL. Across a range of health conditions, symptoms have consistently been shown to be the most important determinants for HRQoL and well-being [2]. Symptoms such as pain, tiredness, depression and anxiety often co-occur and affect multiple dimensions of life [10], underscoring the need for a more symptom-focused approach in both research and practice.

By examining the prevalence of different symptoms and their association to HRQoL, areas for targeted interventions and policy adjustments can be identified to enhance the well-being of older adults, thereby reducing potential healthcare burdens and improving societal wellbeing [2].

The aim of this study was to investigate the prevalence of nine core symptoms (pain, tiredness, sleepiness, nausea, loss of appetite, depression, worry/anxiety, shortness of breath, and overall well-being) among older people in the population and how these symptoms relate to the HrQoL.

## Methods

### Study design and population

This was a cross-sectional analysis of the population-based VAScular and Chronic Obstructive Lung Disease (VASCOL) study of 73-year-old men [9]. The VASCOL study is an ongoing longitudinal cohort study of older men’s health that started in 2010 as part of a screening campaign for abdominal aortic aneurysms offered to all men aged 65 years in the County of Blekinge, Sweden. VASCOL has been detailed elsewhere [9]. Data for the current study were collected at a follow-up in 2019, when the participants were approximately 73 years old, using a postal survey that was sent out to participants who were still alive with a known address. The follow-up postal survey focused on lifestyle, symptoms, health conditions, and well-being. Inclusion criteria for this study were complete data on the measurements used. Participants in the VASCOL cohort were similar to Swedish reference for men in the same age concerning current smoking habits, height, lung function, and civil status. BMI values and education level were higher than the reference values [9].

### Assessments

Self-reported height and weight were used to calculate the body mass index (BMI, kg/m^2^). Education level was the self-reported highest level completed (elementary school, upper secondary school, professional school, or university), categorised as university degree or no university degree for the analysis.

Physical activity was self-reported in categories of frequency (less than once a week, 1–3 times a week, or 3–6 times a week or every day) and exertion (sedentary, moderate exercise, moderate but regular exercise, and frequent exercise). Participants’ smoking history was self-reported and calculated as pack-years of tobacco smoking. The following physician-diagnosed conditions were also self-reported: chronic obstructive pulmonary disease (COPD), asthma, tuberculosis, sleep apnoea, or other lung diseases), myocardial infarction, angina, atrial fibrillation, heart failure, valvular heart disease, coronary artery bypass grafting, aortic aneurysm, carotid artery stenosis, and stroke.

To measure the experience of the nine core symptoms (pain, tiredness, sleepiness, nausea, loss of appetite, depression, worry/anxiety, shortness of breath, and overall well-being), the Swedish validated version of the Edmonton Symptom Assessment Scale, revised version (ESAS-r) was used [11]. Each symptom on the ESAS-r can be scored on an 11-point numeric rating scale (NRS), ranging from 0 (“no symptom”) to 10 (“worst possible severity”). Additional measures for anxiety and depression included the Swedish validated version of the Hospital Anxiety and Depression Scale (HADS) [12]. The HADS comprises 14 items each scored on a 4-point (0–3) scale which can be summarised to an anxiety and depression domain score (range 0–21), respectively. A higher score means more severe anxiety/depression. Participants were asked about their experience with the symptoms during the last 2 weeks. Presence of symptoms was defined as a score ≥ 1 for the symptoms measured by ESAS-r as this cut-off corresponds to the established minimum clinically important difference (MCID) [13]. For the HADS, a score of > 8 on either the anxiety or depression subscale was considered indicative of clinically relevant symptoms, consistent with established cut-offs [14].

Physical and mental HRQoL was evaluated using the Swedish validated version of the Short-Form 12 Health Survey version 2 (SF-12v2) [4] which comprises of 12 items which are calculated into separate physical and mental scores. Questions regarding the physical HrQoL score include limitations in activities, accomplishments, and pain interference, while the mental HrQoL score pertains to questions on accomplishments, energy, mood, and social time [4]. A higher score reflects better physical/mental HrQoL.

### Statistical analyses

Participants characteristics were summarised using descriptive statistics.

Associations between the symptoms and physical and mental HRQoL were analysed using linear regression, unadjusted and adjusted for the following potential confounders: BMI, education level (university degree or not), physical activity (frequency and exertion), pack-years of smoking, and the presence of cardiovascular and respiratory diseases. Strengths of association were reported as beta coefficients with 95% confidence intervals (CIs). Statistical analyses were conducted using SPSS version 27(SPSS Chicago, Il. USA, 2020).

### Ethical considerations

The study protocol was approved by the Swedish Ethical Review Authority (DNr: 2019 − 00134) and all participants gave written informed consent.

## Results

Out of 1900 invited (all men), 1302 men participated in the VASCOL baseline. At the time of the follow-up in 2019, a total of 106 had deceased and 3 had unknow address. A total of 907 replied to the follow-up survey and were included in the present study. Their mean age was 73.0 (standard deviation [SD]: 0.7) years and the average BMI was 27.1 (SD: 3.8), Table 1. A substantial proportion of participants reported one or more respiratory diseases (16%) and cardiovascular diseases (35%). The median number of pack-years of smoking was 5.5 (IQR: 0 to 12.2). The education level varied, with more than one-fifth (22%) of participants having university education. One third of participants (31%) engaged in some form of physical activity 3–6 times a week, with moderate intensity being the most common exertion level (62%).

**Table 1:** – Characteristics of 907 men aged 73-years in the population

Variable (% complete data)	
Age (100%)	73.22 (0.67)
BMI (99%)	27.12 (3.81)
Pack-years of smoking (97%)	5.47 (0-12.17)
One or more respiratory diseases (100%)*	143 (16)
One or more cardiovascular diseases (100%)**	318 (35)
Post-high school education (94%)	191 (22)
Physical activity, frequency (99%)	
Less than once a week	136 (15)
1–3 times a week	254 (29)
3–6 times a week	276 (31)
Every day	228 (26)

*Includes chronic obstructive pulmonary disease (COPD), asthma, tuberculosis, sleep apnea, or other lung diseases

**Includes myocardial infarction, angina, atrial fibrillation, heart failure, valvular disease,coronary artery bypass grafting, aortic aneurysm, carotid artery stenosis, stroke. All numbers are n (%), except for pack-years of smoking which is median (interquartile range), and age and body mass index (BMI) which are mean (standard deviation)

*BMI* Body mass index

The most prevalent physical symptoms (any severity, score ≥ 1/10) were tiredness (reported by 78% of participants, with a mean severity score of 2.4 [SD: 2.3]), poor well-being (77%; mean severity score 2.4 [SD: 2.3]), and pain (73%; mean severity score of 2.5 [SD: 2.5]) (Table 2). Nausea and lack of appetite were less prevalent, reported by approximately one-fifth of participants, with lower mean severity scores of 0.5 (SD: 1.3) and 0.5 (SD: 1.4), respectively (Table 2).

Psychological symptoms assessed included depression and anxiety, measured using different tools with distinct thresholds (≥ 1 for ESAS-r and ≥ 8 for HADS), reflecting different constructs (Table 2). Depression measured by ESAS-r was reported by 46% of participants, with a mean score of 1.2 (SD: 1.9), while clinically relevant depression measured with HADS (score > 8) was found in 9% of participants, with a mean score of 3.0 (SD: 3.1) Anxiety measured by ESAS-r was reported by 32% of participants, with a mean score of 0.9 (SD: 1.7); and clinically relevant anxiety by HADS was found in 12% of participants, with a mean score of 3.4 (SD: 3.4).

**Table 2:** – Prevalence and severity of symptoms in 73-year-old men in the population

Symptom (% complete data)	Presence of symptom (score ≥ 1/10), *n* (%)	Mean severity score (SD)
Tiredness (97%)	689 (78%)	2.42 (2.27)
Poor well-being (97%)	679 (77%)	2.38 (2.30)
Pain (98%)	650 (73%)	2.51 (2.45)
Drowsiness (96%)	614 (70%)	2.03 (2.13)
Shortness of breath (98%)	493 (56%)	1.78 (2.33)
Depression (98%)	407 (46%)	1.20 (1.91)
Anxiety (98%)	287 (32%)	0.86 (1.72)
Other symptoms (93%)	338 (40%)	1.27 (2.10)
Nausea (98%)	191 (22%)	0.49 (1.29)
Lack of appetite (98%)	183 (21%)	0.54 (1.44)
HADS Anxiety (96%)	107 (12%)	3.39 (3.43)
HADS Depression (96%)	76 (9%)	2.99 (3.08)

Symptoms are listed in descending order of prevalence. For symptoms measured with the Edmonton Symptom Assessment Scale – revised (ESAS-r), presence was defined as a score >1 on an 11-point numeric rating scale (NRS), ranging from 0 (“no symptom”) to 10 (“worst possible severity”). For the Hospital Anxiety and Depression Scale (HADS), the presence of clinically relevant depression or anxiety was defined as a domain score of >8. “Other symptoms” were reported in a free-text field (e.g constipation) and included symptoms not listed among the core ESAS-r items

The associations between symptoms and HRQOL are presented both unadjusted and adjusted for confounders in Table 3. The associations between symptoms and HrQoL were similar in both models. As symptom severities worsened, both the physical and mental HrQoL scores also worsened. A similar pattern was seen across all the evaluated symptoms. Notably, the three symptoms showing the strongest associations with physical HrQoL, after adjusting for the confounders, were pain, tiredness, and shortness of breath (Table 3). For mental HrQoL, the symptoms with strongest adjusted associations were depression, anxiety, tiredness, and drowsiness (Table 3).

**Table 3:** – Association between symptoms and physical and mental HrQoL

Symptom (% complete data)	Beta coefienct (CI) physical HrQoL (unadjusted)	Beta coefienct (CI) physical HrQoL (adjusted)	Beta coefienct (CI) mental HrQoL (unadjusted)	Beta coefienct (CI) mental HrQoL (adjusted)
Tiredness (97)	-2.31 (-2.54 to -2.09)	-1.98 (-2.22 to -1.74)	-2.14 (-2.37 to -1.92)	-2.30 (-2.56 to -2.03)
Poor well-being (97)	-1.44 (-1.70 to -1.19)	-0.87 (-1.12 to -0.61)	-1.78 (-2.02 to -1.54)	-1.70 (-1.97 to -1.43)
Pain (98)	-2.44 (-2.64 to 2.25)	-2.05 (-2.25 to -2.24)	-1.10 (-1.34 to -0.86)	-1.11 (-1.38 to -0.86)
Drowsiness (96)	-2.03 (-2.29 to -1.77)	-1.66 (-1.93 to -1.40)	-2.31 (-2.56 to -2.07)	-2.35 (-2,64 to -2.07)
Shortness of breath (98)	-2.24 (-2.45 to -2.02)	-1.73 (-1.97 to -1.49)	-1.14 (-1.39 to -0.90)	-1.19 (-1.49 to -0.90)
Depression (98)	-1.41 (-1.72 to -1.10)	-0.85 (-1.18 to -0.51)	-3.52 (-3.73 to -3.30)	-3.50 (-3.74 to -3.27)
Anxiety (98)	-1.19 (-1.54 to -0.85)	-1.19 (-1.54 to -0.85)	-3.39 (-3.65 to -3.12)	-3.40 ( -3.70 to -3.11)
Other symptoms (93)	-1.15 (-1.44 to -0.86)	-0.99 (-1.27 to -0.72)	-1.34 (-1.62 to -1.06)	-1.33 (-1.64 to -1.02)
Nausea (98)	-1.99 (-2.46 to -1.52)	-1.43 (-1.90 to -0.96)	-2.77 (-3.22 to -2.33)	-2.74 (-3.25 to -2.23)
Lack of appetite (98)	-1.88 (-2.31 to -1.45)	-1.37 (-1.80 to -0.95)	-3.09 (-3.48 to -2.70)	-3.05 (-3.49 to -2.61)
HADS Anxiety (96)	-0.74 (-0.92 to -0.57)	-0.56 (-0.73 to -0.39)	-1.90 (-2.02 to -1.77)	-1.93 (-2.08 to -1.79)
HADS Depression (96)	-0.94 (-1.13 to -0.74)	-0.66 (-0.86 to -0.47)	-1.92 (-2.07 to -1.77)	-2.03 (-2.20 to -1.86)

Associations between symptoms and health-related quality of life (HRQoL), presented as beta coefficient (95% CI). Both unadjusted and adjusted regression results are shown for physical and mental HRQoL outcomes. Adjusted for body mass index (BMI), education level, physical activity, smoking history and cardiovascular and respiratory disease. Symptoms are listed in descending order of prevalence

## Discussion

### Main findings

This study demonstrates a considerable symptom burden in this population sample of older men, with multiple symptoms being both highly prevalent and strongly associated with poorer physical and mental HRQoL. Pain, tiredness and drowsiness were among the most commonly reported symptoms and showed particularly strong associations with lower HRQoL. Depression and anxiety were also prevalent and clearly linked to both physical and mental aspects of quality of life. Notably, these associations remained significant even after adjusting for relevant confounders such as BMI, education level, physical activity, smoking history and the presence of cardiovascular and respiratory diseases, indicating that the symptom burden is independently linked to lower HRQoL.

These findings support earlier studies showing that symptoms - regardless of underlying diagnoses - can strongly impact perceived health and everyday functioning in older individuals [15]. The high prevalence of both physical and mental symptoms in this age group emphasises the complexity of aging and reinforces the need for a more symptom-oriented and well-being approach to health in older populations. By studying older men specifically, the results also contribute new knowledge about a group that is often under-represented in health research, despite known differences in help-seeking behaviour and symptom expression [9].

### What this study adds

This study delineates the prevalence of different symptoms and how these symptoms relate to HRQoL in a population sample of older men. Most previous research has primarily examined how chronic diseases affect HRQoL, while some studies have focused on the role of individual symptoms irrespective of the underlying conditions [16]. By analysing symptoms independently of diagnoses, this study provides a more nuanced understanding of some of the factors influencing HRQoL in older men.

Notably, this study also contributes to the growing evidence that mental health symptoms, such as depression and anxiety, are as strongly associated with HRQoL in a similar way to physical symptoms such as pain and tiredness [6, 15]. Given that men may be less likely to report psychological distress or seek help for mental health concerns [7], these findings highlight the importance of targeted screening and intervention strategies. Additionally, the study strengthens previous indications that symptom burden itself - rather than the presence of specific diseases - may be a more important and direct predictor of HRQoL [2, 15].

### Implications

Clinicians and policymakers may benefit from greater awareness of symptom burden and its association with HRQoL. From a clinical perspective, the results suggest that older individuals - regardless of diagnosis - may experience a range of symptoms that affect daily functioning and overall well-being. Yet, for clinicians, the symptoms (or at least some of them, such as breathlessness) can often be secondary to the illness and as such under-explored [17]. Equally, individual symptoms in older, frailer individuals can be dismissed as part of general co-morbidity and, as such, may be ignored unless the burden becomes critical [17]. Symptoms can be normalised as part of ageing and therefore their impact might not be volunteered by the individuals themselves; this can be further underscored by individuals’ stoic approach to health issues, where symptom burden is only mentioned when it becomes extreme [17, 18]. This underlines the importance of person-centred and comprehensive approaches to assessment and response, especially in primary care, where the focus lies not only on diagnosing disease but also on understanding the patient’s lived experience of symptoms. Instruments such as ESAS-r and HADS can support systematic identification of individuals at risk of lower HRQoL, even when no clear underlying disease is present.

On a societal level, the findings highlight the need for health promotion and preventive efforts that consider both physical and mental health in older men. Public health initiatives targeting symptom relief - particularly in the areas of pain, tiredness, depression and anxiety - may help reduce the burden on healthcare systems and support aging populations in maintaining independence and quality of life. Social prescribing which links primary care with community support may also be beneficial for improving health and wellbeing, including anxiety/depression, though more robust research and evaluation is needed to ensure its effectiveness [19, 20]. Such public initiatives are especially important in light of demographic changes, where a growing proportion of the population is entering old age [1].

### Strengths and limitations

The use of validated instruments in a large, age-homogenous cohort enhances the reliability of the findings. The fact that all participants were 73-year-old men reduces variation due to age, allowing a focused analysis of symptom burden and its association with HRQoL. However, the sole inclusion of men of the same age prevents generalisability to women or other age groups. All data were self-reported, which may introduce recall or reporting bias, particularly in relation to sensitive symptoms such as depression and anxiety [21]. Additionally, as the study is cross-sectional, it cannot establish causality between symptoms and HRQoL. Still, the consistency with previous research strengthens the validity of the findings.

## Conclusions

By identifying the symptoms most strongly associated with HRQoL - particularly pain, tiredness, depression and anxiety - this study offers guidance for targeted clinical screening and public health efforts in the population of older men. A more proactive and individualised approach to symptom management may promote healthier aging and improved well-being in an increasingly ageing population.

## Data Availability

The dataset used in this study is available from the corresponding author upon reasonable request. New study objectives must be approved by the Sweden’s national ethical review board.

## Abbreviations

BMI: Body mass index
COPD: Chronic obstructive pulmonary disease
CI: Confidence interval
ESAS-r: Edmonton Symptom Assessment Scale, revised version
HADS: Hospital Anxiety and Depression Scale
HrQoL: Health-related quality of life
IQR: Interquartile range
MCID: Minimum clinically important difference
NRS: Numeric rating scale
SD: Standard deviation
SF-12v2: Short-Form 12 Health Survey
VASCOL: VAScular and Chronic Obstructive Lung Disease study
